# Cholangiocarcinoma presenting as a solitary epididymal metastasis: a case report and review of the literature

**DOI:** 10.1186/1746-1596-2-33

**Published:** 2007-08-30

**Authors:** Victoria S Bennett, David M Bailey

**Affiliations:** 1 Department of Cellular Pathology, John Radcliffe Hospital, Headley Way, Headington, Oxford, OX3 9DU, UK; 2 Department of Cellular Pathology, Wycombe General Hospital, Queen Alexandra Road, High Wycombe, Buckinghamshire, HP11 2TT, UK

## Abstract

**Background:**

Solid tumor metastasis to the epididymis is a rare occurrence and is mostly discovered incidentally at autopsy or after therapeutic orchidectomy for prostate cancer. Other primary carcinomas that have been demonstrated to metastasize to the paratesticular region include those originating in the stomach, kidney, ileum, and colon.

**Case presentation:**

A 72-year-old gentleman presented with a firm and tender mass involving the right epididymis. On examination, he was jaundiced. Computed tomography of the abdomen demonstrated an obstructive stricture of the extra-hepatic bile ducts, in keeping with a cholangiocarcinoma, through which a metal stent was endoscopically inserted for symptomatic relief.

Subsequent right radical orchidectomy yielded a diffusely infiltrative adenocarcinoma obliterating the epididymis, extending into the rete testis, vas deferens and spermatic cord and showing widespread vascular and perineural invasion. Residual epididymal, rete, and testicular tubules showed no in situ neoplasia. Morphologically and immunohistochemically the features were in keeping with a metastasis from a primary cholangiocarcinoma.

**Conclusion:**

Only two cases of bile duct carcinoma metastasising to the male genital tract have previously been reported in the literature, the testis being the main site of metastasis in both cases. To our knowledge, this is the first described case of cholangiocarcinoma metastasising primarily to the epididymis, and presenting as a solitary epididymal metastasis in the absence of disseminated disease. It serves to highlight the importance of performing a thorough examination of the male external genitalia both clinically, in the follow up of cancer patients, and at autopsy.

## Background

Solid tumor metastasis to the testis and paratesticular region is uncommon and principally encountered as an incidental autopsy finding [1]. Metastasis to the epididymis is relatively rare, fewer than fifty cases having been described in the published literature to date. The majority of such metastases are of prostatic origin and found incidentally at therapeutic orchidectomy [2]. We report a case of metastatic cholangiocarcinoma presenting as an epididymal mass; to our knowledge the first case described in the English literature.

## Case presentation

A 72-year-old gentleman presented with pain and swelling around the right testis. On admission, he was jaundiced. Scrotal examination revealed a firm and tender mass, separate from the testis, in the region of the epididymis and distal spermatic cord. Ultrasound examination showed prominence and thickening of the right epididymis, normal right testicular appearances, and a right hydrocele. Computed tomography demonstrated marked dilatation of the intrahepatic biliary tree extending distally through the extrahepatic ducts to the confluence of the left and right hepatic ducts. The common hepatic and bile ducts were not clearly seen although a subtle infiltrate encasing the vessels at the porta hepatis was noted. No other significant pathology affecting the thorax, abdomen, or pelvis was seen. Serum bilirubin and alkaline phosphatase were elevated in keeping with the obstructive picture. Endoscopic retrograde cholangio-pancreatography revealed a long and irregular stricture of the common bile duct extending into the common hepatic duct. A 150 mm metal stent was inserted to relieve the obstruction through which bile was subsequently seen to drain effectively. A clinical and radiological diagnosis of cholangiocarcinoma was made.

Subsequently, an uncomplicated right inguinal orchidectomy was undertaken.

## Pathology

**Figure 1:**
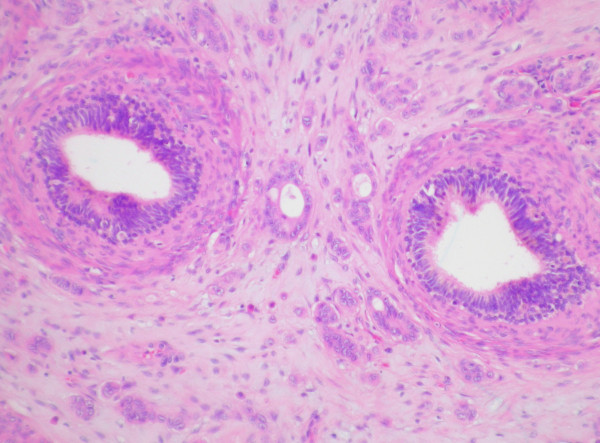
Haematoxylin and eosin stained section showing the tumour infiltrating around and into the epididymal tubules, which themselves show no in situ neoplastic change.

**Figure 2:**
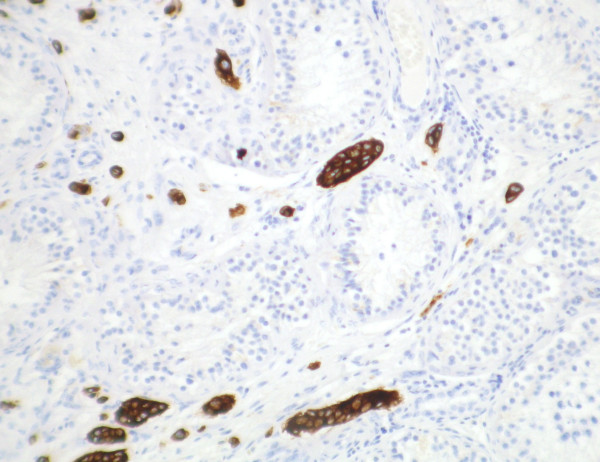
Immunoperoxidase staining for CK7 highlighting the interstitial growth pattern of the tumour within the testicular parenchyma.

Macroscopically, the epididymis was obliterated by a firm white mass (40 × 15 × 13 mm), which extended into the rete testis and spermatic cord and displaced the adjacent testis. Histologically, the tumour was composed of epithelial cells showing a focal columnar morphology, forming glands, trabeculae, and cords, together with scattered individual discohesive cells, set within an eosinophilic spindle cell stroma. The tumour cells showed moderate nuclear pleomorphism and frequent mitotic figures. The lesion was diffusely infiltrative, extending through the epididymis (fig 1) and focally into the rete testis, vas deferens and spermatic cord. There was very focal interstitial infiltration into the adjacent testicular parenchyma sparing the seminiferous tubules. Where present, residual epididymal, rete, and testicular tubules were of normal appearance, showing no in situ neoplasia. Widespread vascular and perineural invasion by tumour was present. Immunohistochemically, the tumour cells showed positivity for EMA, CEA, Ca125, and CK7 (fig 2) and were negative for CK20, PSA, PAP, calretinin, and thrombomodulin. Strong cytoplasmic diastase resistant PAS positivity was also present.

The appearances were those of an adenocarcinoma. The differential diagnosis included a primary tumour, arising from the epididymis or rete testis, or a metastasis. In a recent paper, Amin et al [3] described features favouring a metastatic paratesticular tumour including: age greater than fifty years, a clear history of primary malignancy elsewhere, histology not resembling a known, or common, primary testicular or paratesticular tumour, frequent lymphovascular invasion, and an interstitial growth pattern. On that basis, and in view of the absence of in situ neoplasia, a diagnosis of metastatic cholangiocarcinoma was made.

Unfortunately, the patient subsequently developed ascites and died from an upper gastrointestinal bleed.

## Conclusion

It has been noted that 3.6% of all malignant tumours in the testis [4] and 8.1% in the paratesticular region are metastases [5]. They appear to follow a bimodal distribution with leukaemias and small round blue cell tumours being more common in childhood and adolescence, and solid tumours, in particular adenocarcinomas, and lymphomas being more common in older men, usually presenting around the sixth decade. Metastatic tumours to the epididymis are usually identified in the setting of disseminated disease [6] and only uncommonly occur as a solitary site of metastasis. It is even more rare for an epididymal metastasis to present as the first manifestation of an occult primary neoplasm [5,6]. In some cases, metastasis to the testis or paratesticular tissues is the first sign of tumour recurrence [7], often following definitive treatment. This highlights the importance of examining the external genitalia in the follow up of all male cancer patients.

In 1925 Henke and Lubardch were the first to describe epididymal metastasis from a renal cell carcinoma [8]. Subsequently, in 1927, Keifer noted epididymal spread from a primary pancreatic carcinoma found incidentally at autopsy [9]. Since then, up to 50 cases of solid tumour metastases to the epididymis, with or without spermatic cord involvement, have been recorded [2]. Many of these were discovered incidentally at autopsy or on examination of orchidectomy specimens removed therapeutically for prostate cancer. The former observation reinforces the importance of performing a thorough examination of the male external genitalia at autopsy and the latter explains, in part, why the prostate has thus far proven to be the most common primary site for epididymal metastasis from epithelial tumours (37%) [8]. Other common primary sites include stomach (18%), kidney (16%), colon (13%), ileum, particularly carcinoid tumours, (8%), and pancreas (5%) [8]. Some recent papers, particularly from Japan, suggest that gastric adenocarcinoma is beginning to supersede prostatic adenocarcinoma as the most common primary site for epididymal metastases [3,10] This may be consequent upon the decline in popularity of orchidectomy as a therapeutic modality in prostate cancer with the advent of other less invasive treatments such as hormonal manipulation.

Proposed routes of metastasis to the epididymis, particularly in the case of a prostatic primary, include direct extension from adjacent organs and intraductal spread via the vas deferens. Other proposed mechanisms, more likely in the case that we describe (particularly in view of the extensive vascular invasion present within and surrounding the tumour), include retrograde venous and lymphatic extension, and arterial embolism.

On review of the literature, we found only two cases of bile duct carcinoma metastasising to the male genital tract [1,6], the testis being the main site of metastasis in both cases. To our knowledge, ours is the first case of epididymal metastasis originating from a primary cholangiocarcinoma of the extrahepatic bile ducts presenting as a solitary epididymal metastasis in the absence of disseminated disease.

## Abbreviations

EMA- Epithelial membrane antigen.

CEA- Carcinoembryonic antigen.

Ca125- Cancer antigen 125.

CK- Cytokeratin.

PSA- Prostate specific antigen.

PAP- Prostate acid phosphatase.

PAS- Periodic acid Schiff.

## Competing interests

The author(s) declare that they have no competing interests.

## Authors' contributions

VB drafted the manuscript with assistance from DB. Both authors read and approved the final manuscript.
